# Subject-specific tribo-contact conditions in total knee replacements: a simulation framework across scales

**DOI:** 10.1007/s10237-023-01726-1

**Published:** 2023-05-21

**Authors:** Benedict Rothammer, Alexander Wolf, Andreas Winkler, Felix Schulte-Hubbert, Marcel Bartz, Sandro Wartzack, Jörg Miehling, Max Marian

**Affiliations:** 1 Engineering Design, Friedrich-Alexander-Universität Erlangen-Nürnberg (FAU), Erlangen, Germany; 2 Department of Mechanical and Metallurgical Engineering, School of Engineering, Pontificia Universidad Católica de Chile, Santiago, Chile

**Keywords:** Total knee replacement, Biomechanics, Biotribology, Multiscale simulation, In silico modeling

## Abstract

Fundamental knowledge about in vivo kinematics and contact conditions at the articulating interfaces of total knee replacements are essential for predicting and optimizing their behavior and durability. However, the prevailing motions and contact stresses in total knee replacements cannot be precisely determined using conventional in vivo measurement methods. In silico modeling, in turn, allows for a prediction of the loads, velocities, deformations, stress, and lubrication conditions across the scales during gait. Within the scope of this paper, we therefore combine musculoskeletal modeling with tribo-contact modeling. In the first step, we compute contact forces and sliding velocities by means of inverse dynamics approach and force-dependent kinematic solver based upon experimental gait data, revealing contact forces during healthy/physiological gait of young subjects. In a second step, the derived data are employed as input data for an elastohydrodynamic model based upon the finite element method full-system approach taking into account elastic deformation, the synovial fluid’s hydrodynamics as well as mixed lubrication to predict and discuss the subject-specific pressure and lubrication conditions.

## Introduction

Besides other etiologies such as postoperative infections (Sundfeldt et al. 2006), the long-term performance of biomedical endoprostheses like total knee replacements (TKRs) and the associated risk for painful and costly complications such as tissue inflammation, osteolysis, and premature prosthesis failure are highly driven by the wear resistance of the articulating surfaces (Shah et al. 2021). Generally, TKRs consist of a hard femoral component, usually ceramics or metals such as cobalt-chromium or titanium alloys, rubbing against the conformal and soft bearing surface of a polymeric tibial inlay, mostly ultrahigh molecular weight or highly cross-linked polyethylene (UHMWPE, HXLPE). A distinction can be made between implants with a round-on-round and a flat-on-flat design (Wirtz 2011). The former exhibit a high degree of interlocking and constrain to the degrees of freedom due to the curvature of both the femoral and tibial components in the anterior–posterior (AP) and in the medial–lateral (ML) direction. Flat-on-flat designs are the standard inlay of many current prostheses and only feature curvatures in the AP direction while the components are flat in the ML direction as well as parallel to the transverse plane. In addition to macro-geometry as well as material and surface properties of the implant components, the overall biotribological performance is particularly affected by the prevailing stress and lubrication conditions during the complex and dynamic activities (Ruggiero et al. 2018, 2020a; Nečas et al. 2021b; Marian et al. 2021; Ruggiero 2020). For designing and optimizing TKR components with respect to their macro-geometry or respective surface modifications such as coatings or textures (Rothammer et al. 2021a, 2021c; Marian et al. 2022a; Tremmel et al. 2020), fundamental knowledge about in vivo contact forces and relative velocities at the articulating interfaces as well as present deformations, contact pressures, stresses, and lubrication conditions is essential. It is well accepted that wear phenomena observed after retrieval of TKR do not match the ones from components tested in joint simulators following the ISO 14243-3 (ISO—International Organization for Standardization 2014) gait cycle (Harman et al. 2009; Orozco Villaseñor and Wimmer 2016), suggesting that testing standards might not adequately mimic the actual in vivo conditions (Lundberg et al. 2012). Thereby, in silico numerical modeling could promote in-depth knowledge and enhance the development and performance prediction of improved TKRs, which cannot be precisely determined using conventional (in vivo) measurement methods (Bergmann et al. 2014). Thus, the increasingly relevant stress spectra of younger patients (Sundfeldt et al. 2006), who usually lead a more active and joint-loading lifestyle (Liu et al. 2015), can be considered, deviating from the loads and kinematics standardized in ISO 14243-3. Respective findings can also be utilized to define experimental testing conditions for characterizing TKRs in component or model tests (Rothammer et al. 2021a; Affatato and Ruggiero 2019).

In silico modeling of TKRs generally covers the domains of biomechanical musculoskeletal simulation (Askari and Andersen 2021a, 2021b) as well as tribo-contact simulation to allow for a stringent representation of the loads during a gait cycle from the macro to the microscale (Affatato and Ruggiero 2020). Musculoskeletal simulations utilize multibody biomechanical human models and inverse kinematic and dynamic simulation approaches in order to compute joint kinematics, joint torques, muscle activations, muscle forces, or joint reaction forces from experimental motion data (Damsgaard et al. 2006). Typically, multibody human models are composed of rigid bodies connected with mechanical joints, representing the passive locomotor apparatus, as well as muscle–tendon models, representing the active locomotor apparatus (Damsgaard et al. 2006). Using force-dependent kinematics (Andersen et al. 2009, 2010, 2017), these multibody models can be supplemented with detailed artificial joint models to compute joint kinematics as well as muscle, ligament and contact forces (Marra et al. 2018, 2015). Moissenet et al. (2017) and recently Tomasi et al. (2022) systematically reviewed the state-of-the-art for estimating hip and knee joint loads through musculoskeletal modeling. Thereby, several studies, such as those by Askari and Andersen (2021a; b) or Affatato and Ruggiero (2019), Ruggiero and Sicilia (2020a, 2021; b), Ruggiero et al. (2018, 2020b), demonstrate that musculoskeletal simulations of artificial joints can directly be coupled to or generate input data for contact models.

Regarding numerical approaches to study the tribo-contacts in synovial joints, models based on the finite element method (FEM) assuming dry conditions have been widely developed (Donahue et al. 2002; O’Brien et al. 2015; Penrose et al. 2002) and even partly coupled to musculoskeletal multibody-dynamics (Shu et al. 2018; Hua et al. 2022) as well as wear predictive models (Zhang et al. 2017). However, similar to natural joints, it became evident that the lubrication with synovial fluid (SF) affects the biotribological behavior and the complex interplay between the SF hydrodynamics and rheology in combination with elastic deformations of the bearing surfaces have to be taken into account (Nečas et al. 2021a, b; Gao et al. 2022). This is usually referred to as elastohydrodynamic lubrication (EHL). Mattei et al. (2011), Nečas et al. (2021a) and Gao et al. (2022) recently reviewed numerical analyses of hip and knee replacements. Various methods have been developed to solve and couple the system of equations consisting of the Reynolds equation representing the hydrodynamic pressure build-up, the elasticity, the lubricant gap, and the load balance equations. Due to the more complex geometries and kinematics and the fact that hard-on-soft pairings have to be considered, TKRs are less studied compared to total hip replacements (THR), where hard-on-hard pairings have numerically been investigated so far. While initial studies were limited in terms of geometry, kinematics, and stress collectives, Su et al. (2011) developed a time-dependent EHL model based upon the multi-grid finite difference approach to solve the Reynolds differential equation for the fluid hydrodynamics coupled with the calculation of the elastic deformation using a constrained column model. Thereby, it was demonstrated that squeeze effects allow to maintain a fluid film during the stance phase of gait while the entrainment velocity was largely responsible for forming the film formation in the swing phase. Moreover, it was shown that surface design parameters like the conformity of the surfaces could be optimized to increase the lubricant gap. Gao et al. (2017, 2018) employed a numerical model based upon the multi-grid (MG) and the spherical fast Fourier transformation (SFFT) method while accounting for mixed lubrication and shear thinning effects of the synovial fluid to predict the wear behavior. Butt et al. (2021) presented a mixed lubrication model, which considered the complex geometry of the human knee implant. It was reported that the lubrication regime varied between elastohydrodynamic, mixed and boundary and that multiple points of contact can occur per condyle during the gait cycle. Recently, we (Marian et al. 2021) adapted the full-system FEM approach (Habchi 2018a), taking into account mixed lubrication, time-transient and thermal effects as well as non-Newtonian fluid behavior. Most notably, the approach was directly validated against optical fluorescent measurements on a knee simulator (Nečas et al. 2021b) and recommendations for the complexity of the numerical model were derived (Marian et al. 2021). The aforementioned works (Gao et al. 2018; Butt et al. 2021; Marian et al. 2021) have in common that they assumed the ISO gait cycle (ISO—International Organization for Standardization 2014) and that a round-on-round TKR configuration was studied while, to our best knowledge, flat-on-flat configurations in combination with more realistic physiological stress spectra differing from the ISO standard have not been addressed so far.

To summarize, in silico modeling with a bidirectional or unidirectional coupling of biomechanical musculoskeletal with simulation of the lubricant tribo-contacts in TKRs allows for a prediction of the loads, velocities, deformations, stresses, and lubrication conditions across the scales during a gait cycle. In principle, there is still relatively little work in this direction for lubricated hard-on-soft pairings of TKRs. In addition, reported studies focus on elderly patients or standardized motion and round-on-round designs, while the stress spectra for the increasingly relevant group of younger subjects as well as the behavior of flat-on-flat designs have received little attention to date. Within the scope of this contribution, we therefore employ state-of-the-art musculoskeletal modeling to compute relative knee velocities as well as contact forces in the medial and lateral TKR contacts of flat-on-flat TKRs by means of inverse dynamics approach and force-dependent kinematic solver based upon experimental gait data measured of young persons in a motion capture laboratory. These data are further applied to numerical EHL simulations in order to predict the subject-specific lubrication conditions, contact pressures, deformations, and stresses. It is noteworthy that both, the musculoskeletal and the contact simulation, is implemented using commercial multiphysics software solutions which facilitates reconstruction and utilization without the need to implement complex solution algorithms. Thus, we hope to further stimulate and accelerate the research and optimization in the biomechanical and biotribological behavior of artificial synovial joints such as TKRs. In the following, the theory and governing algorithms or equations for the musculoskeletal biomechanical simulation as well as the tribo-contact simulation are addressed step by step in Sects. 2.1 and 2.2, respectively. The obtained results on contact forces and relative kinematics (3.1) as well as contact and lubrication conditions (3.2) are subsequently presented and discussed in Sects. 3 and 4.

## Material and methods

### Musculoskeletal biomechanical simulation

**Fig. 1:**
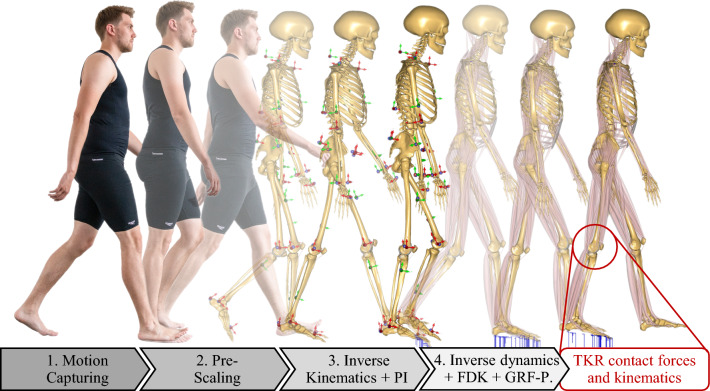
Steps of the musculoskeletal simulation in order to compute contact forces and relative knee kinematics in the medial and lateral TKR contacts

In order to compute contact forces and relative knee kinematics in the medial and lateral TKR contacts using musculoskeletal simulation, the methodology depicted in Fig. 1 was employed. The first step was the measurement of gait data in the motion capture laboratory. The AnyBody Modeling System (AMS) (Damsgaard et al. 2006) was used as musculoskeletal modeling framework. In the second step, a generic baseline musculoskeletal model was pre-scaled to a subject-specific model using the length-mass scaling algorithm (Damsgaard et al. 2006). In the third step, inverse kinematics (Damsgaard et al. 2006) was performed to transfer the measured motion onto the musculoskeletal models. This was done by defining virtual markers on the exact boney landmark positions of the pre-scaled baseline model on which the markers had been attached to the subjects. By applying a least-square optimization approach, the measured marker trajectories were fitted onto the virtual markers by altering the generalized coordinates. Inverse kinematics was performed using the parameter identification (PI) algorithm by Andersen et al. (2010). Thus, the pre-selected marker positions and segment lengths were optimized based on the trajectory data and manually measured subject anthropometry. Subsequently, the baseline musculoskeletal model was anthropometrically scaled to three subject-specific models of the subjects S1–S3. In addition, the measured motions were transferred to the respective subject-specific models and described by generalized coordinates time series.

The last step contained the dynamic analysis of the motion using inverse dynamics (Damsgaard et al. 2006), which employed a muscle recruitment optimization approach to compute muscle forces, joint reactions forces and joint moments. Since no force plates could be used in the experimental setup, the dynamic simulation was conducted using the ground reaction force (GRF) prediction algorithm from Skals et al. (2017). A TKR model was added to the three subject-specific musculoskeletal models using the methodology introduced by Marra et al. (2015) as well as the force-dependent-kinematics (FDK) approach presented by Andersen et al. (2017). FDK is an enhanced inverse dynamic approach, which allows for the simultaneous computation of joint internal forces and secondary kinematics. The approach assumes that the secondary degrees of freedom are not influenced by the global dynamics. Hence, FDK solves for a quasi-static equilibrium between ligaments, muscles, contact forces (between the different TKR parts) and external loads. This modeling technique permitted to realize a musculoskeletal TKR model and to compute contact forces and relative knee kinematics as a part of the inverse dynamic simulation.

**Fig. 2:**
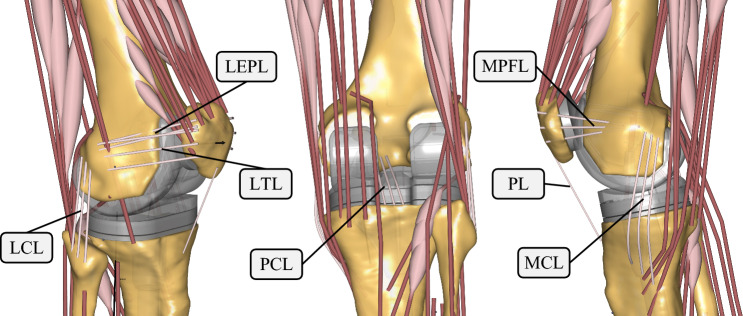
Final FDK TKR model with 11 degrees of freedom and a medial and lateral contact modeled as rigid-rigid contact formulation. The lateral collateral ligament (LCL), lateral epicondylopatellar ligament (LEPL), lateral transverse ligament (LTL), posterior cruciate ligament (PCL), medial patellofemoral ligament (MPFL), patellar ligament (PL) and medial collateral ligament (MCL) were positioned according to Marra et al. (2015)

The motion capturing was conducted using a Qualisys motion capture system with nine cameras of the type Oqus 300 + at 60 Hz. A total of 38 reflective markers were attached to the subjects, according to the plugin-gait marker protocol. Three healthy subjects (all male, age 25–26 years, body height 1.73, 1.75, and 1.96 m, body weight (BW; 63.5, 70.0, and 103.0 kg) volunteered for the motion capture study. The subjects performed multiple gait cycles without interruption on a treadmill at a constant speed of 1.1 m/s. Additionally, the anthropometry of the subjects was measured. The marker trajectory data were postprocessed, labeled and exported using the Qualisys Track Manager 2.11. The FullBody_GRFPrediction-Model from the AnyBody Managed Model Repository 2.1. served as the baseline musculoskeletal model. The leg models were replaced by the Twente Lower Extremity Model Version 2.0 (TLEM 2.0) (Carbone et al. 2015). The TKR model was realized using STL-data of the femur’s, tibia’s and fibula’s bony morphology as well as the STL-endoprosthesis of the sixth grand challenge competition to predict in vivo knee loads (Fregly et al. 2012). Correct subject-specific TKR modeling included the correct scaling of the TKR parts (STL-Files) in respect to the bones using scale matrices deduced from the scaling process. The correct TKR positioning, the muscle attachment points, the degrees of freedom as well as the ligaments’ positions, stiffnesses and reference strains were modeled for each subject-specific model according to the methodology of Marra et al. (2015). Additionally, the wrapping surfaces were lengthened to prevent muscles from “snapping over” after scaling. Furthermore, the reference stress of the medial patellofemoral ligament (MPFL) was changed from 0.08 to 0.03 as indicated by Chen et al. (2014). The final TKR model is representatively shown for one subject in Fig. 2.

The simulations were performed for eight succeeding gait cycles per subject. The muscles in the musculoskeletal model were modeled utilizing the three-element hill-type muscle model (Zajac 1989). Inverse dynamics was performed using polynomial muscle recruitment of the fifth order. Six residual forces and moments were defined at the pelvis, in order to balance possible dynamic inconsistencies. The FDK simulation was performed using the internal stiffness values, the pressure module, the step interval and the FDK residual forces as recommended by Marra et al. (2015). By applying this methodology, the medial and lateral contact forces *F*_med_ and *F*_lat_, the center of pressure as well as the relative knee kinematics could be computed in the medial and lateral TKR contact for three subjects and their respective motion data. Using the relative knee kinematics and the information of the center of pressure over time, the TKR contact velocities of the lateral and medial tibia part *u*_tib,med_ and *u*_tib,lat_ as well as the lateral and medial femur part *u*_fem,med_ and *u*_fem,lat_ were determined.

The musculoskeletal simulations were evaluated with respect to their kinematic error as well as their residual forces and moments. The lowpass filter threshold applied to the motion data (15 Hz), the marker positions (subject-specific), the marker weights of the parameter identification (subject-specific), the parameters of the GRF-prediction method (parameters proposed by Skals et al. (2017)) as well as the muscle recruiting approach (to the fifth order) were optimized in order to achieve the best possible results regarding these values. Additionally, the TKR modeling parameters were optimized by using a reference musculoskeletal model, which was scaled using the body height and weight data of the sixth grand challenge to predict in vivo knee loads (Fregly et al. 2012), in order to compare the in vivo measured results of the challenge with our simulations when using the motion and GRF data provided by the challenge. In addition, the final results of our simulation, i.e. the total contact forces computed using the three subject-specific musculoskeletal models, were compared to the extreme values of the in vivo measured total contact forces by Bergmann et al. (2014) as a plausibility check of the general evolution and magnitude of the predicted results due to the lack of publicly available data suitable for verification or validation.

### Tribo-contact simulation

N﻿umerical modeling of the elastohydrodynamically lubricated (EHL) contacts in the medial and lateral condyle between the femoral and tibial component was done by means of the full-system FEM approach (Habchi et al. 2007) using the software Comsol Multiphysics. Thereby, the obtained evolution of forces and velocities during the gait from the musculoskeletal simulation were used as tabular inputs. Essentially, the implementation followed our earlier work (Marian et al. 2021). However, in contrast to the round-on-round design studied therein, the flat-on-flat configuration studied within the scope of this contribution was interpreted as a line contact with a length *l* of 28.3 mm for the medial and 27.3 mm for the lateral compartment as well as no curvature in ML direction as determined from the TKR components’ STL-data of the sixth grand challenge. The hydrodynamics of the synovial fluid were described by means of the Reynolds differential equation

1 $$\underbrace {{\frac{\partial }{\partial x}\left( {\frac{{\rho \cdot h^{3} }}{12 \cdot \eta }\frac{\partial p}{{\partial x}}} \right)}}_{{{\text{Poiseuille}}\;{\text{term}}}}\underbrace {{\frac{\partial }{\partial x}\left( {\rho \cdot h \cdot u_{{\text{m}}} } \right)}}_{{{\text{Couette}}\;{\text{term}}}}\underbrace {{\frac{\partial }{\partial t}\left( {\rho \cdot h} \right)}}_{{{\text{Squeeze }}\;{\text{term}}}} = 0,$$

with the coordinate *x*, the hydrodynamic pressure *p*, the lubricant gap *h*, the viscosity *η*, the density *ρ*, the effective velocity *u*_m_, and the time *t*. In line with the findings from our previous study (Marian et al. 2021), tibial rotation as well as piezo-viscous and thermal effects were neglected while time-transient effects were considered. The fluid was assumed to have a density of 1 000 kg/m^3^ and a viscosity of 0.1 Pa∙s, which are representative values for patients suffering from diseased joints at higher shear rates (Marian et al. 2021; Rothammer et al. 2021b; Mazzucco et al. 2002). Cavitation effects were addressed by a penalty formulation (Wu 1986; Marian et al. 2017). The fluid film equation

2 $$h = h_{0} + \frac{{x^{2} }}{2 \cdot R} + \delta$$

includes the rigid body distance *h*_0_, a quadratic approximation of the undeformed geometry and the elastic deformation *δ* (normal displacement). The equivalent radius

3 $$R = \frac{1}{{\frac{1}{{R_{{\text{f}}} }} + \frac{1}{{R_{{\text{t}}} }}}}$$

was derived from the femoral radius *R*_f_ (= 38 mm) and the tibial radius *R*_t_ (= 58 mm) in anterior–posterior direction as determined from the STL-data. The deformation was calculated for an equivalent body with equivalent Young’s modulus

4 $$E = \frac{{E_{{\text{f}}}^{2} \cdot E_{{\text{t}}} \cdot \left( {1 + \upsilon_{{\text{f}}}^{2} } \right)^{2} + E_{{\text{t}}}^{2} \cdot E_{{\text{f}}} \cdot \left( {1 + \upsilon_{{\text{t}}}^{2} } \right)^{2} }}{{\left[ {E_{{\text{f}}} \cdot \left( {1 + \upsilon_{{\text{t}}} } \right) + E_{{\text{t}}} \cdot \left( {1 + \upsilon_{{\text{f}}} } \right)} \right]^{2} }}$$

and equivalent Poisson’s ratio

5 $$\upsilon = \frac{{E_{{\text{f}}} \cdot \upsilon_{{\text{t}}} \cdot \left( {1 + \upsilon_{{\text{t}}} } \right) + E_{{\text{t}}} \cdot \upsilon_{{\text{f}}} \cdot \left( {1 + \upsilon_{{\text{f}}} } \right)}}{{E_{{\text{f}}} \cdot \left( {1 + \upsilon_{{\text{t}}} } \right) + E_{{\text{t}}} \cdot \left( {1 + \upsilon_{{\text{f}}} } \right)}}$$

by employing the linear elasticity equation

6 $$\nabla \left[ {C \cdot \varepsilon \left( U \right)} \right] = 0,$$

where *C* is the generalized Hooke’s law elasticity matrix, *ε* is the contracted Lagrangian small-strain tensor, and *U* is the displacement vector. Thereby, Young’s moduli of 240,000 MPa (*E*_f_) and 660 MPa (*E*_t_) as well as Poisson’s ratios of 0.29 (*ν*_f_) and 0.46 (*ν*_t_) were assumed in accordance with Marian et al. (2021), representing a cobalt-chromium femoral and an UHMWPE tibial component, respectively.

**Fig. 3:**
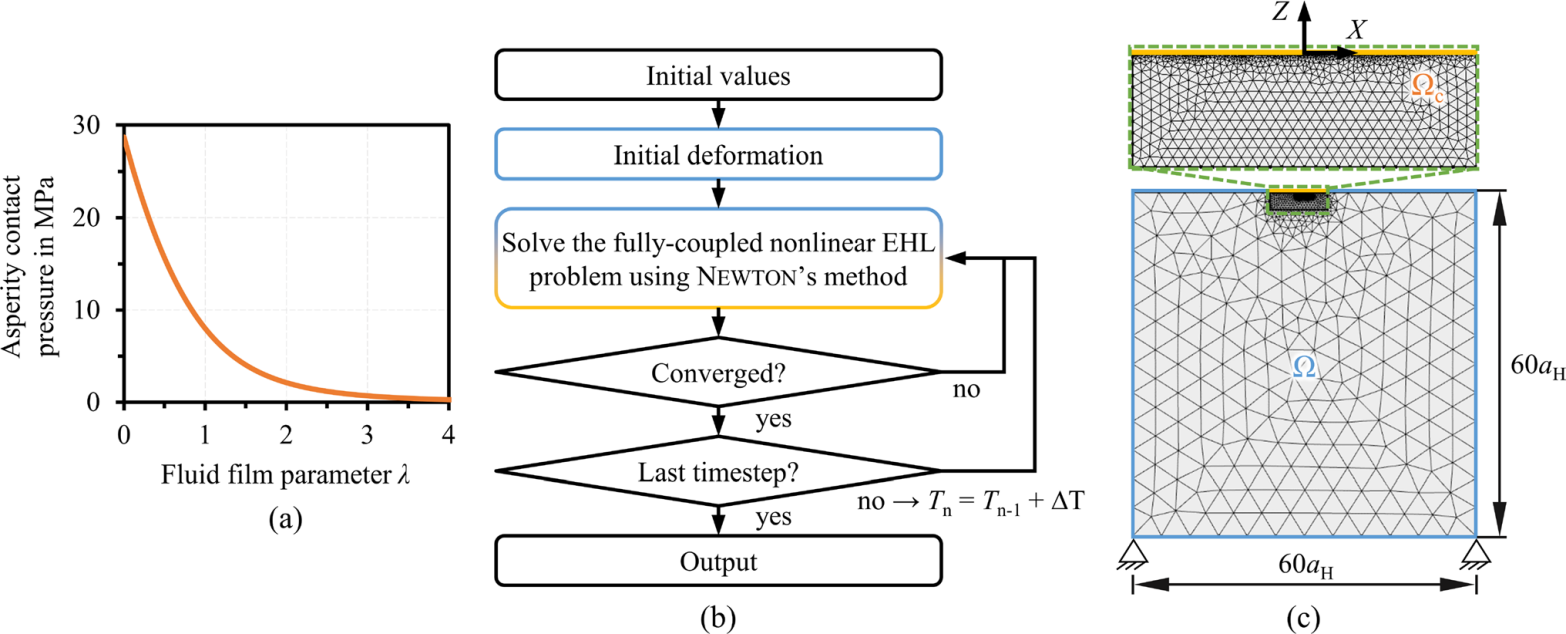
**a** Solid asperity pressure graph, **b** numerical solution scheme, and **c** FEM-domain

Mixed lubrication effects, i.e. solid asperity contact of the rubbing surfaces, were considered by means of a stochastic coupling with the macro-EHL model. The asperity contact pressure was calculated by a statistical Greenwood-Tripp model (Greenwood and Williamson 1966; Greenwood and Tripp 1970) under the assumption of linear elasticity and a Gaussian distribution. Therefore, the roughness parameters of actual TKA components complying to ISO 7207-2 (ISO–International Organization for Standardization 2014) were analyzed by laser scanning microscopy (VK-X200, Keyence) and a combined root mean squared roughness *σ* of 1.95 μm was determined for the calculations. The implementation in MathWorks MATLAB followed Winkler et al. (2020) and the obtained solid asperity pressure *p*_a_ versus the fluid film parameter

7 $$\lambda = \frac{h}{\sigma }$$

was incorporated into the macro-scale EHL model as an interpolated function, see Fig. 3a).

The integral over the total pressure balanced the normal load to satisfy the equilibrium of forces:

8 $$\mathop \smallint \limits_{{{\Omega }_{{\text{c}}} }} \left( {p + p_{{\text{a}}} } \right){\text{d}}x = \frac{F}{l}$$

To ensure good conditioning and convergence, the relevant variables were normalized on Hertzian values (index H) at a reference time step *t*_ref_:

9 $$\begin{aligned} X & = \frac{x}{{a_{{\text{H}}} \left( {t_{{{\text{ref}}}} } \right)}}, Z = \frac{z}{{a_{{\text{H}}} \left( {t_{{{\text{ref}}}} } \right)}}, P = \frac{p}{{p_{{\text{H}}} \left( {t_{{{\text{ref}}}} } \right)}}, P_{{\text{a}}} = \frac{{p_{{\text{a}}} }}{{p_{{\text{H}}} \left( {t_{{{\text{ref}}}} } \right)}}, H = \frac{h \cdot R}{{a_{{\text{H}}}^{2} \left( {t_{{{\text{ref}}}} } \right)}}, \overline{\delta } = \frac{\delta \cdot R}{{a_{{\text{H}}}^{2} \left( {t_{{{\text{ref}}}} } \right)}}, \\ T & = \frac{{t \cdot u_{{\text{m}}} \left( {t_{{{\text{ref}}}} } \right)}}{{a_{{\text{H}}} \left( {t_{{{\text{ref}}}} } \right)}}, \overline{\rho } = \frac{\rho }{\rho } = 1, \overline{\eta } = \frac{\eta }{\eta } = 1. \\ \end{aligned}$$

More information on the dimensionless form and the normalization for transient operating conditions can be found elsewhere (Marian et al. 2021; Raisin et al. 2016, 2015). The overall FEM solution scheme is illustrated in Fig. 3b). After initialization with Hertzian values for the reference time step, the Reynolds equation was solved in a weak form on the contact domain *Ω*_c_ and strongly coupled with the calculation of the elastic deformation of domain *Ω* for first time step. The solution domain depicted in Fig. 3c) was discretized by a free triangular mesh with a refinement in the contact center of the upper surface. Zero pressure (Dirichlet) boundary condition was applied at the in- and outlet of *Ω*_c_. Moreover, zero displacements on the bottom, the total pressure as normal stress on the top, and free boundaries (no normal and shear stresses) on the remaining borders of *Ω* were employed. The contact domains were chosen large enough to avoid numerical starvation and an influence due to limited deformability. The Galerkin least squares method (Brooks and Hughes 1982) and isotropic diffusion (Zienkiewicz et al. 2014) were used for numerical stabilization, whereby care was taken to minimize the influence on the numerical solution. Following convergence of the first time step, the time loop was launched and repeated until the last time step of the gait cycle was simulated. The coupling of two consecutive time steps was realized by an implicit backward differentiation formula scheme of second order. For more details about FEM fundamentals applied to EHL problems and the implementation in Comsol Multiphysics, the interested reader is referred to (Marian et al. 2021, 2019, 2022b; Habchi 2018b).

Finally, we analyzed the solid frictional power

10 $$P_{{\text{f}}} = \mathop \smallint \limits_{t} \mathop \smallint \limits_{{{\Omega }_{{\text{c}}} }} \left[ {\mu \cdot p_{{\text{a}}} \left( {x,t} \right) \cdot l \cdot {\text{d}}x} \right]{\text{ d}}t$$

as the timely and spatial integral of the product of the solid–solid coefficient of friction *μ*, the solid asperity contact pressure, and the contact width over the whole gait cycle. To eliminate the influence of the friction coefficient on the evaluation, the solid friction powers of the different compartments and subjects were compared relatively and normalized to the highest value within the studied data set. This still allows a qualitative assessment of the susceptibility to wear of the TKRs depending on the stress collectives, whereby lower numbers indicate less solid–solid contact, i.e. a higher proportion of a load-carrying SF film, and thus lower wear. (Fig. 3).

## Results

### Musculoskeletal simulation: contact forces and relative kinematics

**Fig. 4:**
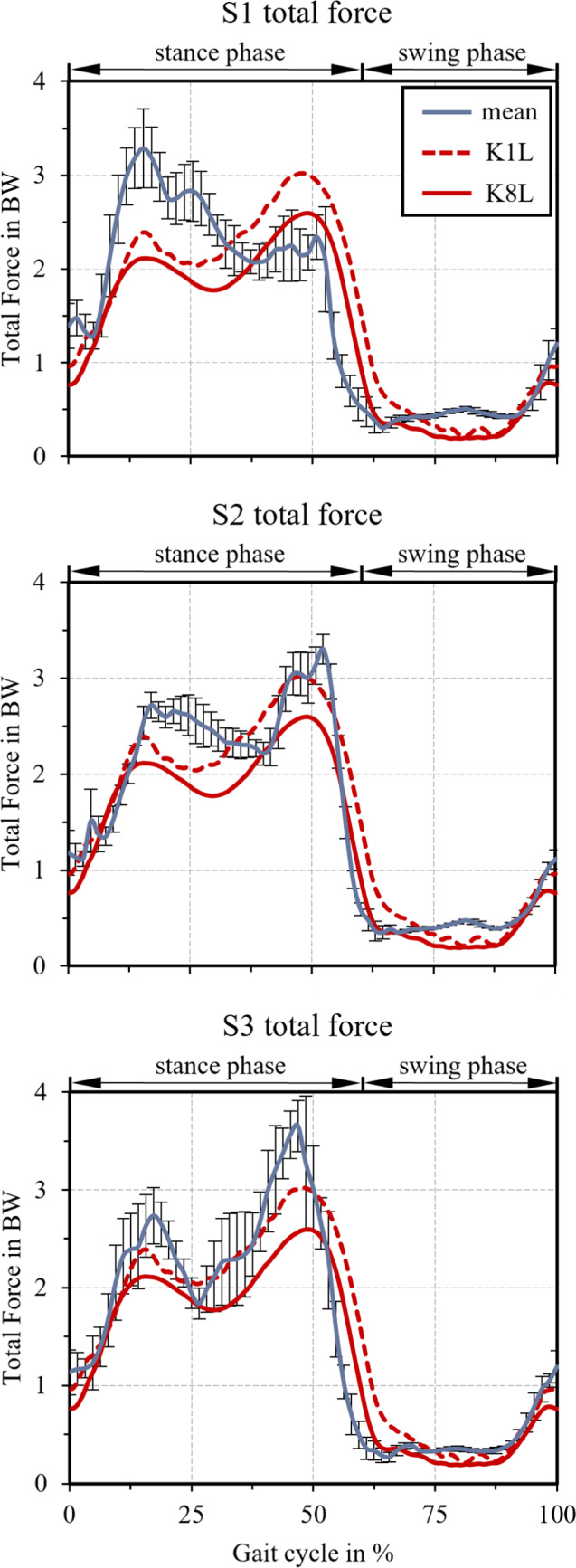
Mean values and standard deviations of the simulated total TKR contact force (in BW) for all three subjects in comparison to the extreme samples of the in vivo measured total contact forces by Bergmann et al. (2014) (patient K1L and K8L)

Mean residual forces in the musculoskeletal simulations were below 5 N, max. residual forces below 40 N, mean residual moments below 2 Nm and max. residual moments below 10 Nm. These values indicate a high consistency of the performed simulations with the measured motion data and good conformity of the FDK model. The kinematic errors in the FDK simulations smaller than 10^–6^ m for all simulations. The residual forces and moments also remained low for all eight gait cycles (S1: 0.91 N mean, 37.52 N max, 0.26 Nm mean, 9.72 Nm max; S2: 1.06 N mean, 12.77 N max, 0.33 Nm mean, 3.19 Nm max; S3: 4.10 N mean, 27.43 N max, 1.19 Nm mean, 7.54 Nm max). The mean of the simulated total TKR contact force (in BW) of the final simulations for all three subjects in comparison to the extreme results of the in vivo measured total contact forces by Bergmann et al. (2014) (patient K1L and K8L) is depicted in Fig. 4. It should be emphasized that this serves for a plausibility check of the obtained results while a direct comparison of our data and Bergmann et al. (2014) is not fully conclusive since different subjects (young subjects vs. actual TKR patients) are underlying the employed data. The computed total TKR load graphs showed the characteristic two-peak curve at the start and the end of the stance phase as observed by Bergmann et al. (2014) and Marra et al. (2015). The first peak raises up to roughly 3.2 BW for S1, 2.7 BW for S2 and 2.6 BW for S3, while the second peak raises up to 2.4 BW for S1, 3.3 BW for S2 and 3.7 BW for S3. Thus, the first peak is higher than the second peak in case for S1, which is contrary to the graphs of S2 and S3 as well as the data of Bergmann et al. (2014).

**Fig. 5:**
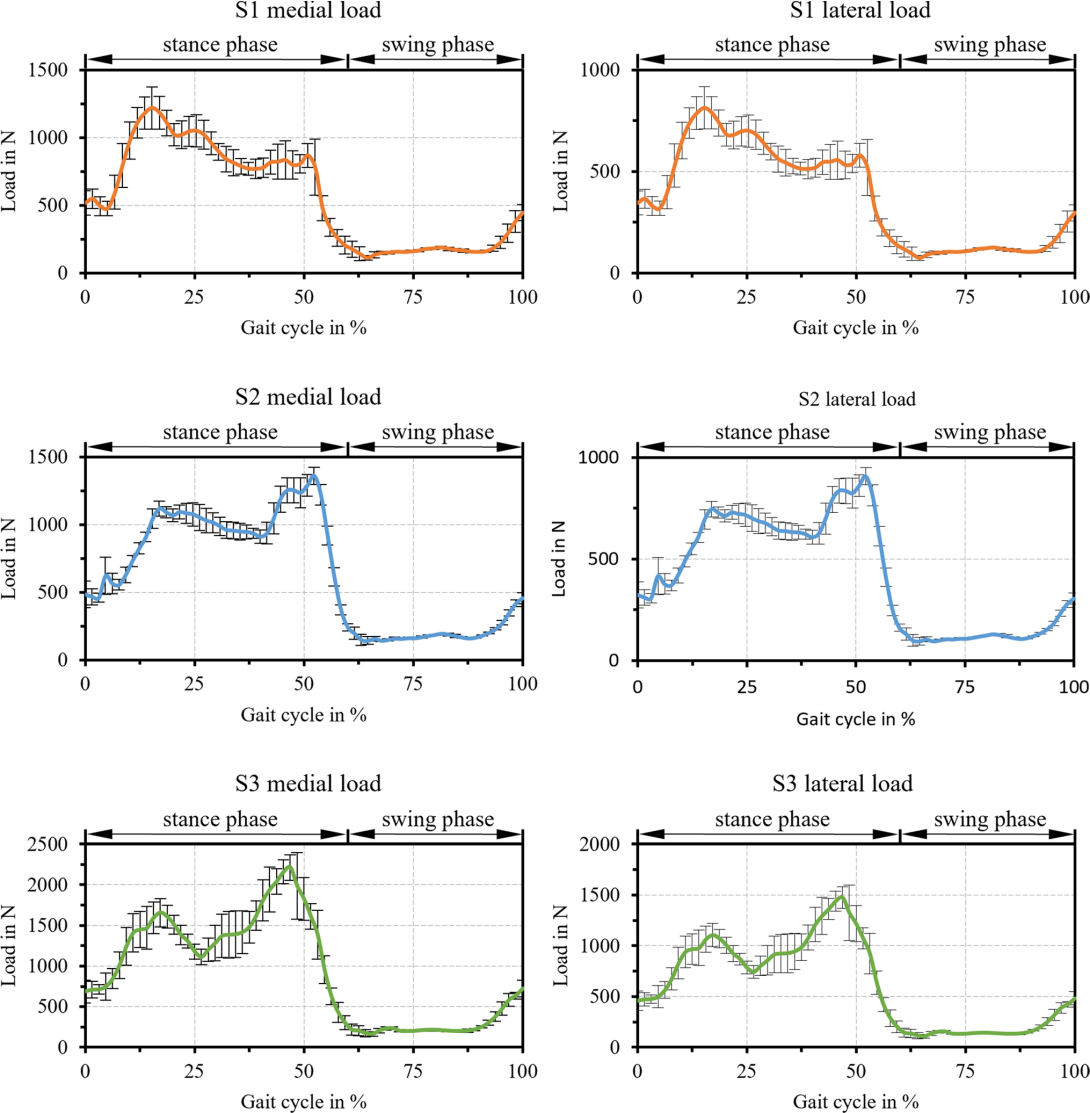
Mean values and standard deviation of the simulated loads in the medial and lateral TKR contact for all eight gait cycles per subject

**Fig. 6:**
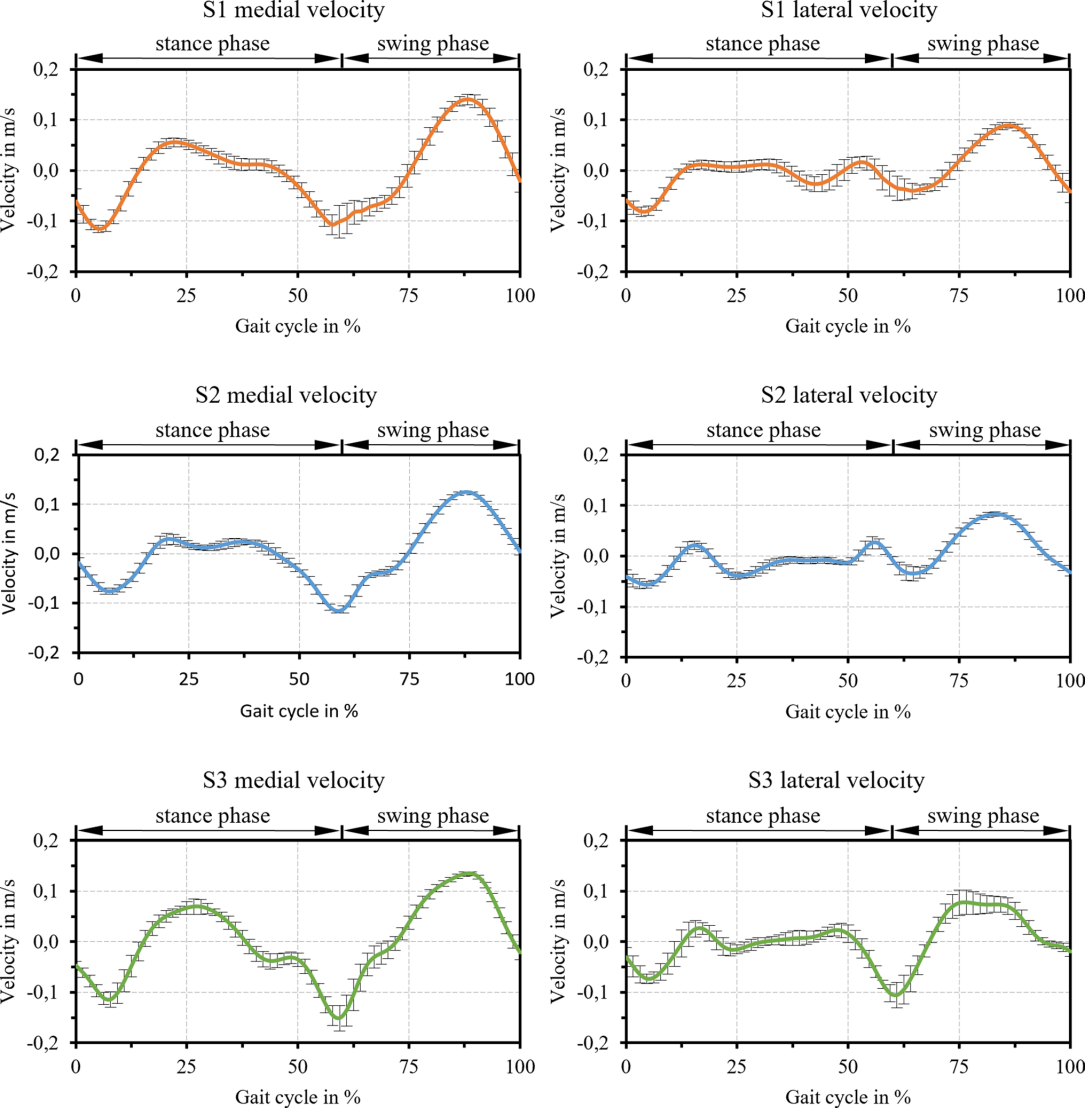
Mean values and standard deviation of the simulated velocities in the medial and lateral TKR contact for all eight gait cycles per subject

The absolute load in the medial and lateral contact for all subjects is displayed in Fig. 5. Hereby, the loads in the medial contact are higher compared to the loads of the lateral contact. This characteristic was also observed by Marra et al. (2015). The contact loads for S3 are comparably higher to S1 and S2, which correlated with the higher body weight of S3. The relative velocity of the medial and lateral contact for all subjects is depicted in Fig. 6. Thereby, the medial contact experiences slightly higher velocities than the lateral contact.

### Contact and lubrication conditions

**Fig. 7:**
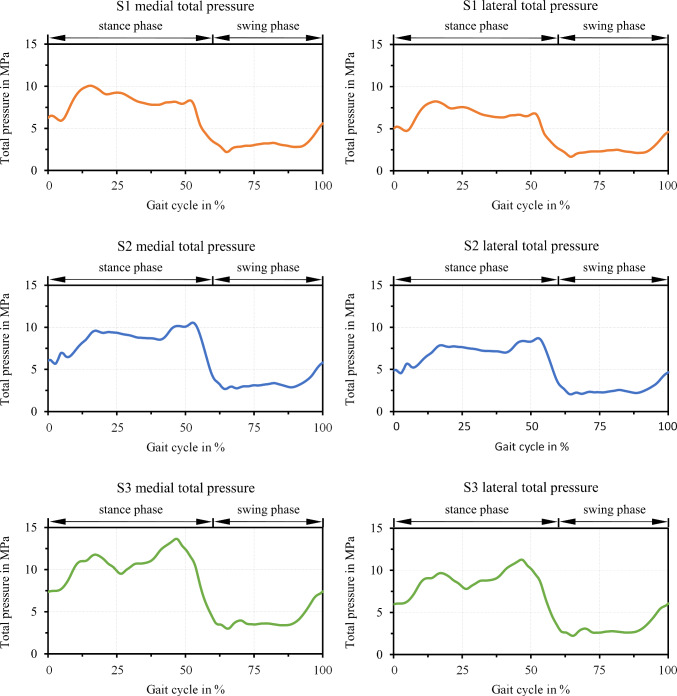
Computed maximum total pressure in the medial and lateral TKR contact over the gait cycle per subject

The computed maximum total contact pressures in the medial and lateral condyle per subject are shown in Fig. 7. Thereby, two peaks in the stance phase at roughly 15% and 50% of the gait cycle could be distinguished that apparently followed the normal force, which indicated that the pressure was mainly generated by the load similar to what would be the case under dry conditions. Accordingly, this was more pronounced for S3 (Fig. 7, green) compared to S2 (blue) and S1 (orange). In the swing phase, the pressure was lower compared to the stance phase and experienced less fluctuations. Nevertheless, two slightly pronounced minima could be seen at around 65% and 90% of the gait cycle, which correlated with the respective normal forces. Generally, the range of the pressure varied between the subjects (S1 medial: 2.2–10.1 Mpa, S1 lateral: 1.7–8.3 Mpa, S2 medial: 2.7–10.5 Mpa, S2 lateral: 2.1–8.7 Mpa, S3 medial: 3.0–13.6 Mpa, S3 lateral: 2.2–11.3 Mpa), with up to 27% difference in between extrema of the different subjects, respectively. Comparing both condyles, the medial compartments experienced up to 22% larger pressures due to the higher normal load proportion.

**Fig. 8:**
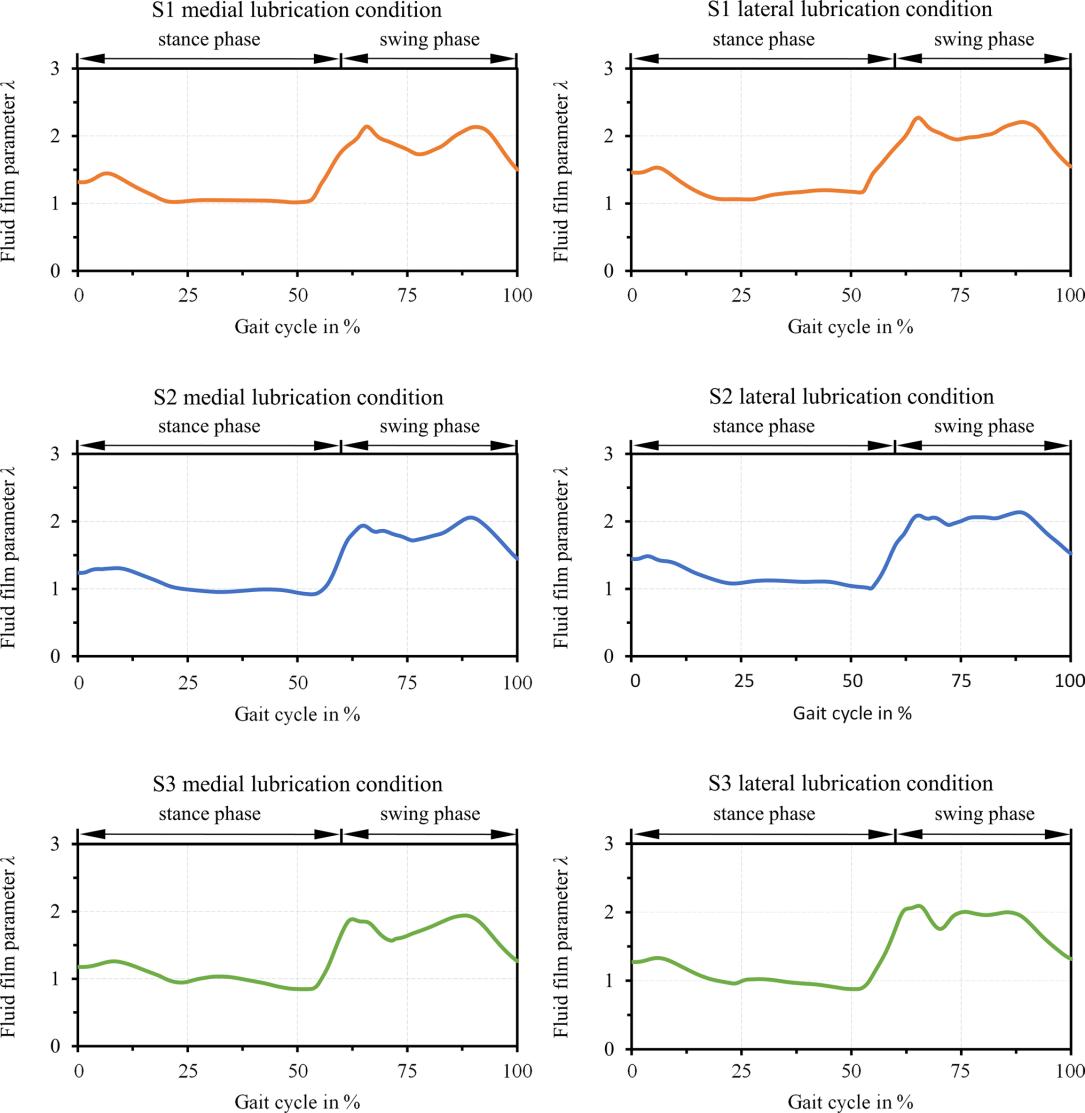
Computed fluid film parameter in the medial and lateral TKR contact over the gait cycle per subject

**Fig. 9:**
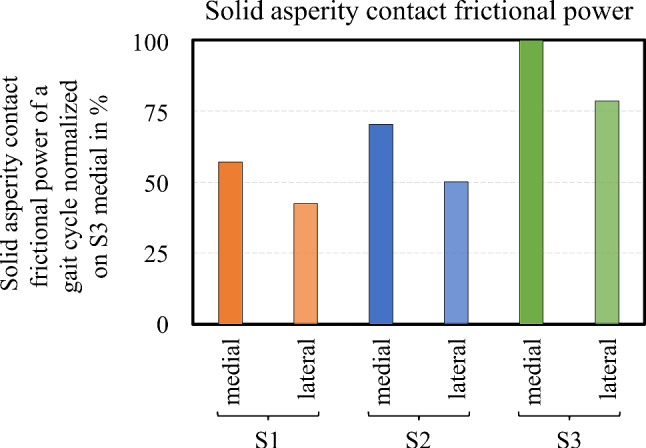
Computed solid asperity contact frictional power of the medial and lateral gait cycle per subject normalized on the maximum value (S3 medial)

The computed fluid film parameter in the medial and lateral condyle per subject depicted in Fig. 8 tended to follow the pressure anti-cyclically and was further influenced by variations in the velocities. Generally, the lubricant gap was smaller in the stance phase due to the higher normal load and the lower entrainment speed. There were two distinct minima in the stance phase at around 20% and 55% of the gait cycle as well as a local minimum at a higher level in the swing phase at 80%, which correlated with pressure peaks and the reversal points of the motion, i.e. zero points of velocity. Two further peaks could be observed in the swing phase around 65% and 90% of the gait cycle due to larger velocities in combination with load minima. Although the three subjects exhibited differences in the load and velocity profiles, similarities were found in the lubrication conditions. The variation between medial and lateral condyle was also not very marked. With lubrication film parameters between one and barely above two, all contacts operated between strong mixed to boundary lubrication (stance phase) to milder mixed lubrication (swing phase). However, the analysis of the solid frictional power presented in Fig. 9 indicated differences between the subjects. The calculated values can be interpreted as an indicator of susceptibility to wear, and it was observed that the lateral condyle accounts for only about 70–75% of the medial compartment. Moreover, S1 and S2 had a 43% and 30% lower solid asperity frictional power than S3, respectively, which is indicative of less wear and can be attributed primarily to the lower load and lower body weight.

## Discussion

The first goal of this study was to provide the necessary input data for EHL simulation by using musculoskeletal modeling and measured motion data. The FDK TKR model was developed using a reference musculoskeletal model using data of the sixth grand challenge to predict in vivo knee loads (Fregly et al. 2012) in conjunction with the knee modeling methodology of Marra et al. (2015) along with their muscle and ligament parameters. The model was adapted to represent the three test subjects of our study. The resulting kinematic errors in the FDK simulations applying the mentioned models were smaller than 10^–6^ m. Mean residual forces in the musculoskeletal simulations were below 5 N, max. residual forces below 40 N, mean residual moments below 2 Nm and max. residual moments below 10 Nm. These values indicate a high consistency of the performed simulations with the measured motion data and good conformity of the FDK model. Bergmann et al. (2014) measured a maximum in vivo contact force of around 3 BW while our simulation results reach up to nearly 4 BW. Our higher values may originate from the age difference and the health conditions of the subjects as well as the fact that our subjects performed the gait barefooted. Nevertheless, the curve progressions show comparable characteristics. Additionally, Marra et al. (2015) used a specific morphing tool to reproduce the musculoskeletal model as precise as possible, making use of the computer tomography data provided by the grand challenge. In comparison, we did not have medical imaging date for the subject-specific musculoskeletal modeling of the subjects’ knees.

The second goal of this study was to use the input data generated from musculoskeletal modeling and measured motion data for subsequent EHL simulation. Thereby, a previously for a round-on-round configuration developed and experimentally validated numerical model (Marian et al. 2021) was adapted for the flat-on-flat design studied within the scope of this article. Thus, it was shown that pressure and lubricant film were driven by the coupled response to the load and velocity profile. Thus, the overall pressures were higher and the lubricant gap smaller during the stance phase compared to the swing phase, which correlates with the reported results from Gao et al. (2017, 2018). Thereby, it was found that the contact pressures were smaller (about half) of that obtained for the round-on-round design assuming an ISO 14243-3 (ISO–International Organization for Standardization 2014) stress collective (Marian et al. 2021; Su et al. 2011). Nevertheless, both the medial and the lateral compartment of all subjects operated in the mixed lubrication regime throughout the whole gait cycle, which contradicts to some extent to the reported film thickness values for an ISO 14243-3 gait cycle reported from Gao et al. (2017, 2018). This means that even younger subjects with active motion profile exhibit considerable risk of wear of TKA wear in the contact area, especially during the strong stressing in the stance phase. However, the swing phase can also be considered wear-inducing due to the solid asperity contact and the lower pressures in the 5 MPa range as lower values have been reported to be even more wear-critical for UHMWPE (Shen et al. 2019; Saikko 2006). In the future, the coupling with wear predictive models adapting the macro- and micro-geometry of the implant components would also be conceivable (Affatato and Ruggiero 2020; Ruggiero et al. 2020b; Ruggiero and Sicilia 2020a, 2020b).

## Limitations

Both, the musculoskeletal multibody as well as the tribo-contact simulations, naturally underlied several assumptions and limitations. First of all, in contrast to Marra et al. (2015), Bergmann et al. (2014), or Chen et al. (2014), we employed healthy subjects for motion capturing. One the one hand, this was intentionally done in order to gain insight into TKR kinematics and dynamics as they would be, if a physiological/pre-implant motion behavior would be regained after implantation. On the other hand, however, this does not allow for a direct verification of the model.

In our study, we replaced the gold standard force plate-based ground reaction force measurement with the prediction method of Skals et al. (2017). This method is validated in the stated publication for various activities, but still has to be validated in the future to pave the way toward clinical applicability. (Ground-truth) force measurements would generally be more valid, but practicability of such approaches is often very restricted due to high pre- and postprocessing effort. Subject-specific simulations based on novel measurement and prediction methodologies, instead of requiring the gold standard of expensive and cumbersome lab-based equipment, such as optical motion capture in conjunction with force plates, would make biomechanical analysis much more accessible to clinical applications.

Within the scope of the numerical tribo-contact modeling, the geometry of the TKR components was simplified and an infinite line contact was assumed. Hence, edge effects or curvatures (radii) at the geometric boundaries of the femur or tibia component were not considered. Even though stress collectives different from the ISO 14243-3 standard could be investigated, the geometric simplification did not permit for consideration of tibial rotation, which might play a more important role in terms of higher shearing differences along the contact width compared to the point-contacts in round-on-round designs. Furthermore, potential hyperelastic material behavior of the UHMWPE was neglected.

The surface roughness parameters were determined from pristine (undamaged and unworn) TKR components. However, the surface roughness might be reduced/smoothed during running-in, thus potentially reducing solid asperity contact (mixed lubrication). Even though the latter was considered, micro-hydrodynamic effects of the surface topography were not taken into account. Additionally, the Reynolds differential equation to describe the SF’s hydrodynamic is subject to various assumptions and might neglect effects due to the somewhat inhomogeneous and colloidal composition of natural SF.

Overall, the models were coupled sequentially, i.e., unidirectionally, and the results from biomechanical simulation were used as input for tribo-contact models. Thus, no direct interaction and feedback of the contact conditions (friction) on the dynamics is considered (Askari and Andersen 2021b). In the future, this might be overcome by directly coupling both modeling approaches, which, however, would potentially result in drastically increased computational time as well as poor convergence. Instead, it would be conceivable to derive analytically solvable proximity equations (Marian et al. 2020) for the consideration of the contact behavior in the biomechanical simulation or, as suggested by Marian et al. (2022b), to train machine learning approaches (Rosenkranz et al. 2021; Marian and Tremmel 2021) that can be employed in higher level multibody simulations.

## Conclusions

Fundamental knowledge about in vivo contact stresses at the articulating interfaces of TKRs are essential for predicting and optimizing the behavior of implant systems. However, the prevailing contact stresses in TKRs cannot be precisely determined using conventional in vivo measurement methods. In silico modeling, in turn, allows for a prediction of the loads, velocities, deformations, stress, and lubrication conditions across the scales during gait. Within the scope of this contribution, we therefore combined musculoskeletal and tribo-contact modeling. In the first step, we computed contact forces with low kinematic errors and very low residual forces and moments. This can be explained by the scope of this study. We wanted to investigate the contact forces during the healthy/physiological gait of young subjects, rather than the best possible reproduction of in vivo measured forces with much older subjects performing unphysiological gait. In a second step, the derived data were employed as input data for an EHL model to predict the subject-specific lubrication conditions, contact pressures, deformations, and stresses. It is particularly noteworthy that both, the musculoskeletal and the contact simulation, were implemented using commercial multiphysics software solutions. Thus, this contribution has the potential to further stimulate and accelerate the research and optimization in the biomechanical and biotribological behavior of artificial synovial joints such as TKRs.

## Data Availability

The data supporting the findings reported in this study are available from B. Rothammer upon reasonable request.

## List of symbols

$a_{{\text{H}}}$: Hertzian contact half-width
$C$: Hooke’s law elasticity matrix
$E$: Equivalent Young’s modulus
$E_{{\text{f}}}$: Femoral Young’s modulus
$E_{{\text{t}}}$: Tibial Young’s modulus
$$F$$: Load
$F_{{{\text{med}}}}$: Medial contact force
$F_{{{\text{lat}}}}$: Lateral contact force
$h$: Lubricant film thickness
$h_{0}$: Film thickness constant parameter
$H$: Dimensionless lubricant film thickness
$l$: Length of contact
$p_{{\text{a}}}$: Asperity contact pressure
$P_{{\text{a}}}$: Dimensionless asperity contact pressure
$p$: Hydrodynamic contact pressure
$P$: Dimensionless hydrodynamic contact pressure
$$P_{{\text{f}}}$$: Frictional power
$$R$$: Equivalent radius
$$R_{{\text{f}}}$$: Femoral radius
$$R_{{\text{t}}}$$: Tibial radius
$$t$$: Time
$t_{{{\text{ref}}}}$: Reference time step
$$T$$: Dimensionless time
$$u_{{\text{m}}}$$: Effective velocity
$u_{{{\text{tib}},{\text{med}}}}$: Velocity of medial tibia part
$u_{{{\text{tib}},{\text{lat}}}}$: Velocity of lateral tibia part
$u_{{{\text{fem}},{\text{med}}}}$: Velocity of medial femur part
$u_{{{\text{fem}},{\text{lat}}}}$: Velocity of lateral femur part
$$U$$: Displacement vector
$$x,z$$: Cartesian coordinates
$X,Z$: Dimensionless Cartesian coordinates
$\delta$: Elastic deformation in z direction
$\overline{\delta }$: Dimensionless elastic deformation in z direction
$\varepsilon$: Contracted Lagrangian small-strain tensor
$\lambda$: Dimensionless film thickness parameter
$\mu$: Coefficient of friction
$\nu$: Equivalent Poisson’s ratio
$\nu_{{\text{f}}}$: Femoral Poisson’s ratio
$\nu_{{\text{t}}}$: Tibial Poisson’s ratio
$$\rho$$: Density
$$\overline{\rho }$$: Dimensionless density
$$\eta$$: Dynamic viscosity
$\overline{\eta }$: Dimensionless dynamic viscosity
$\sigma$: Standard deviation of surface heights
${\Omega }$: Elastic deformation domain
$${\Omega }_{{\text{c}}}$$: Contact domain
